# Cervicovaginal Microbiome and Urine Metabolome Paired Analysis Reveals Niche Partitioning of the Microbiota in Patients with Human Papilloma Virus Infections

**DOI:** 10.3390/metabo10010036

**Published:** 2020-01-15

**Authors:** Nataliya Chorna, Josefina Romaguera, Filipa Godoy-Vitorino

**Affiliations:** 1 Department of Biochemistry, UPR School of Medicine, San Juan 00936, Puerto Rico; 2 PR-INBRE Metabolomics Research Core, UPR School of Medicine, San Juan 00936, Puerto Rico; 3 Department of Ob-Gyn, UPR School of Medicine, San Juan 00936, Puerto Rico; josefina.romaguera@upr.edu; 4 Department of Microbiology & Medical Zoology, UPR School of Medicine, San Juan 00936, Puerto Rico

**Keywords:** cervicovaginal HPV infections, urine, microbiota, multi-omic integrated analyses

## Abstract

In this study, we evaluate the association between vaginal and cervical human papillomavirus infections high-risk types (HPV+H), negative controls (HPV−), the bacterial biota, and urinary metabolites via integration of metagenomics, metabolomics, and bioinformatics analysis. We recently proposed that testing urine as a biofluid could be a non-invasive method for the detection of cervical HPV+H infections by evaluating the association between cervical HPV types and a total of 24 urinary metabolites identified in the samples. As a follow-up study, we expanded the analysis by pairing the urine metabolome data with vaginal and cervical microbiota in selected samples from 19 Puerto Rican women diagnosed with HPV+H infections and HPV− controls, using a novel comprehensive framework, Model-based Integration of Metabolite Observations and Species Abundances 2 (MIMOSA2). This approach enabled us to estimate the functional activities of the cervicovaginal microbiome associated with HPV+H infections. Our results suggest that HPV+H infections could induce changes in physicochemical properties of the genital tract through which niche partitioning may occur. As a result, *Lactobacillus* sp. enrichment coincided with the depletion of *L. iners* and *Shuttleworthia*, which dominate under normal physiological conditions. Changes in the diversity of microbial species in HPV+H groups influence the capacity of new community members to produce or consume metabolites. In particular, the functionalities of four metabolic enzymes were predicted to be associated with the microbiota, including acylphosphatase, prolyl aminopeptidase, prolyl-tRNA synthetase, and threonyl-tRNA synthetase. Such metabolic changes may influence systemic health effects in women at risk of developing cervical cancer. Overall, even assuming the limitation of the power due to the small sample number, our study adds to current knowledge by suggesting how microbial taxonomic and metabolic shifts induced by HPV infections may influence the maintenance of microbial homeostasis and indicate that HPV+H infections may alter the ecological balance of the cervicovaginal microbiota, resulting in higher bacterial diversity.

## 1. Introduction 

Discoveries resulting from human microbiome research have demonstrated that microbes play a role in the susceptibility to HPV and neoplasia [1,2,3]. Oncogenic high-risk human papilloma virus (HPV+H) infections are associated with the development of carcinomas, a malignancy on the rise in multiple populations [4,5,6]. Oncogenic high-risk human papilloma virus (HPV+H) infections are associated with the development of carcinomas, a malignancy on the rise in multiple populations [4,5,6]. Microbes can induce damage to the DNA and lead to mutational events that contribute to cancer. Additionally, mucosal surfaces, vulnerable to environmental disruption, contribute to host-microbial homeostasis, and dysbioses of the microepithelial ecosystem can lead to inflammatory disorders and the progress of malignancies such as cancer [7,8,9,10,11]. The cervicovaginal microbiome has been associated with HPV across ethnicities. Indeed vaginal samples from women of different ethnicities revealed different microbiome signatures, with Hispanics having a higher prevalence of communities not dominated by *Lactobacillus* spp. [12,13]. In Mexican women, *Sneathia spp*. were considered cervical biomarkers for neoplasia [14], while in Puerto Ricans, a loss in Lactobacillus was associated with an increase in *Atopobium vaginae* and *Gardnerella vaginalis* in patients with CIN3 lesions with HPV+H, low-risk HPV, or both co-infections [2]. Cervical microbiota of a majority of HIV negative black South African women was not dominated by Lactobacillus, suggesting that such cervical microbiota may be a contributing factor to the high burden of HIV and HPV infections among black women [15]. Vaginal biomarkers for cervical intraepithelial neoplasia in caucasian, asian, and black women included *Sneathia sanguinegens*, *Anaerococcus tetradius*, and *Peptostreptococcus anaerobius* [1,16]. Another study reported that differences in the diversity in the composition of the cervicovaginal microbiota of asian women are more likely associated with—HPV+H infections. Thus, *Oribacterium*, *Lachnobacterium*, and *Thermus* were associated with HPV 16, while a composition of *Motilibacter* associated with HPV 52, and a composition of *Litorilinea* and *Paludibaculum* with a concomitant paucity of *L. iners* with HPV 58 [17]. Lastly, another study on sex-workers from Western Kenya infected by HPV+H, and some with HIV, showed a significant association between HPV, certain *Candida sp*, and bacterial vaginosis (characterized by a reduction of *Lactobacillus* and overgrowth of anaerobic bacteria) [18]. Most of these findings were based on microbial community structure studies, namely 16S rRNA sequencing, and very few have co-analyzed both microbes and metabolites or metatranscriptomes, especially in paired samples from distant body sites. Given the vast array of newly acquired knowledge in the field and its concomitant technological advances, it is expected that microbial mechanisms underlying several disease states and their metabolites will be discovered. 

The integration of multi-omics techniques such as metagenomics and metabolomics is becoming a very promising approach for the understanding of the complex bacterial interactions with the host and their contributions to systemic health. Given that many metabolites and metabolic pathways are relatively conserved across species, the coupling of two omics approaches provides insights into the association of the human microbiome with health status and risk of disease development [19]. Much work has been done to explore a complex interplay between host-microbiome and metabolome in the gut [20], cervix [21], and saliva [22]. Indeed a recent multi-omic integration study revealed that mainly amino acids and amino acid degradation products, such as polyamines, were well-predicted to be modulated by cervical microorganisms [21].

Most of the studies associating the microbiome and systemic health were conducted via assessment of interactions between the gut microbiome and gut metabolome [23]. A popular strategy now is to explore associations between the gut microbiome and serum metabolome due to the ability of circulating metabolites to translocate through the host’s barriers, and therefore, giving the potential for the identification of systemic health effects [24,25,26,27]. Since metabolites from the gut are absorbed into the circulation and finally excreted through urine [28], recent studies suggest that exploration of the association between urine metabolome and host-microbiome may complement sequencing-based approaches with a functional readout of the gut microbiome [29]. However, a comparison of urine metabolome as a reflection of the serum metabolome was found to be inappropriate since metabolic associations between urine and serum are weaker than those within serum and within the urine, indicating that both data provide independent metabolic information and must be analyzed separately [30]. To our knowledge, there is limited information associated with urine metabolome and cervicovaginal microbiome. Nonetheless, a recent study that applied the integrative multi-omics approach found a complex biochemical interplay between the cervicovaginal microbiome and urine metabolome in women at risk of preterm premature rupture of the membranes [31]. Moreover, this study suggested that monitoring associations between urine metabolome and vaginal microbiota–host interactions could facilitate the stratification of patient groups, which may require or respond appropriately to a given treatment regime. We recently proposed that testing urine as a biofluid could be a non-invasive method for the detection of cervical HPV infections by evaluating the association between cervical HPV types and urinary metabolites [32]. 

As a follow-up study, in the current manuscript, we extend our previous analysis aimed at assessing the possible association of urine metabolome with cervicovaginal microbiota in 19 selected Puerto Rican women. A novel comprehensive framework, the Model-based Integration of Metabolite Observations and Species Abundances 2 (MIMOSA2, borensteinlab.com/software_MIMOSA2.html) [33], was used to systematically link the variance in urine metabolomic data with changes in the composition and structure of communities of cervicovaginal microbiota in the same samples. This approach allowed us to make predictions of the role of microbial taxonomic and metabolic shifts induced by HPV infections on health effects in women at risk of developing cervical cancer. 

## 2. Results

### 2.1. Bacterial Taxa Associated with Vaginal Samples

**Figure 1:**
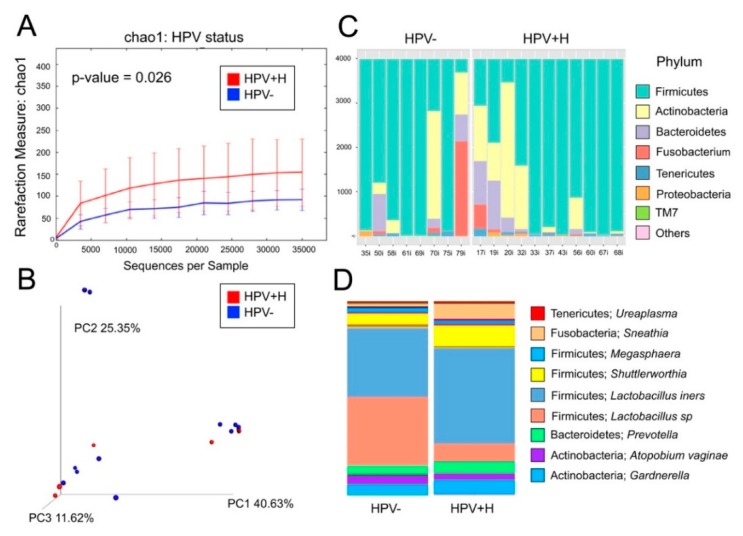
Bacterial community analyses of the 19 vaginal samples. Panel (**A**) depicts rarefaction curves with the Chao1 richness index using a rarefaction level of 35,000 reads; we found that HPV+H were significantly more diverse than HPV− (*p*-value = 0.026). Panel (**B**) shows a 3D beta-diversity plot showing no significant differences in bacterial structure between HPV− and HPV+H. Panel (**C**) shows a phyla-level bar plot showing differences between HPV− and HPV+H. Panel (**D**) shows a bar plot depicting HPV− and HPV+H groups with bacterial taxa at the genus-level.

The selected 19 samples (8 HPV−, 11 HPV+H, Supplementary Table S1), yielded 2,211,246 reads and resulted in 614 OTUs. Data analyses were done using a rarefaction level of 35,000 sequences. We found that vaginal samples from HPV+H had higher bacterial diversity (*p*-value = 0.026) compared to HPV− patients (Figure 1A). In terms of bacterial community structure, no significant differences were found (Figure 1B). HPV+H patients had more bacterial taxa, including Actinobacteria, Bacteroidetes, and a reduction in Firmicutes (Figure 1C). The dominant genus was Lactobacilli, namely *L. iners,* mostly in HPV−, and *Lactobacillus* sp. mostly in HPV+H. *Atopobium vaginae* had a higher abundance in HPV+H and *Shuttelworthia,* and *Sneathia* were more abundant in HPV− (Figure 1D).

### 2.2. Bacterial Taxa Associated with Cervical Samples

**Figure 2:**
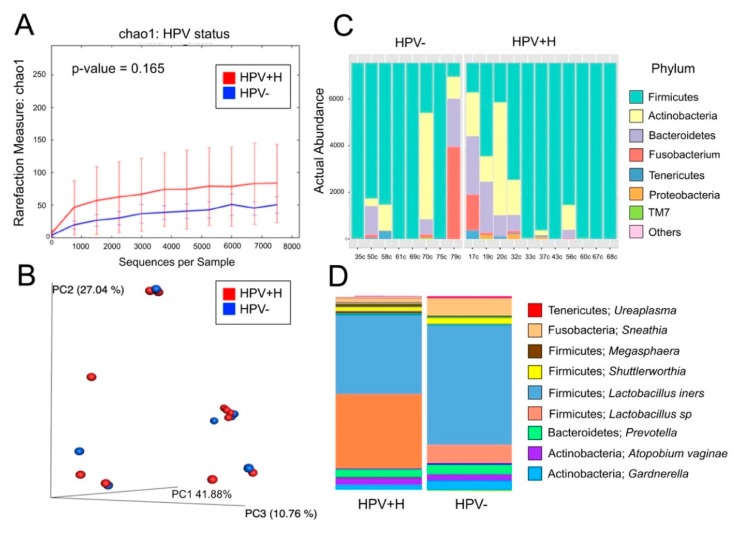
Bacterial community analyses of the 19 cervical samples. Panel (**A**) depicts rarefaction curves with the Chao1 richness index using a rarefaction level of 7500 reads (no significant differences were found). Panel (**B**) shows a 3D beta-diversity plot showing no significant differences in bacterial structure between HPV− and HPV+H. Panel (**C**) shows a phyla-level bar plot showing differences between HPV− and HPV+H. Panel (**D**) shows a bar plot depicting HPV− and HPV+H groups with bacterial taxa at the genus-level.

Regarding the cervix, these 19 samples yielded 1,891,217 reads and resulted in 479 OTUs. Data analyses were done using a rarefaction level of 7500 sequences. We found no significant differences in alpha or beta diversity between HPV+H to HPV− (Figure 2A,B). In terms of bacterial composition, the results were very similar to the vaginal samples with dominant Firmicutes, and a higher abundance of mostly Actinobacteria (Figure 2C). At the genus-level, similar profiles were found in the vaginal samples, where the dominant genus was the Lactobacilli, namely *L. iners,* mostly in HPV−, and *Lactobacillus* sp. *Sneathia* was slightly more abundant in HPV− (Figure 2D).

### 2.3. Association of Urine Metabolites with the Shift in Cervicovaginal Microbiome Induced by HPV+H Infections 

**Figure 3:**
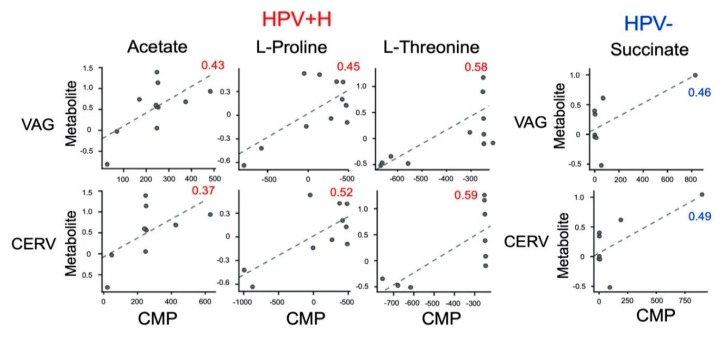
Well-predicted urine metabolites identified by the community metabolic profile (CMP) model in HPV+H and HPV− groups.

As with the microbiota analyses, for the metabolomic analyses, we divided our sample groups of 19 patients (both vagina (introitus) (VAG) and cervical (CERV)) regarding HPV genotyping into HPV− infections (*n* = 8 patients) and exclusively HPV+H types consisting of *n* = 11 patients. Our previous metabolomic analysis showed an altered urine metabolome in the HPV+H group compared to HPV− [32]. Using functional characterization of the VAG and CERV microbiomes with host-circulating urine metabolites, we expected to find more information regarding the extent to which the altered VAG and CERV microbiomes were associated with circulating metabolites in the host’s urine. Therefore, we paired metabolomics [32] and metagenomics data obtained from the same patients using MIMOSA2 [33] and analyzed the two datasets separating HPV− and HPV+H patient samples. To evaluate the relative ability of members of the cervicovaginal microbial community in each sample groupings to produce or utilize individual metabolites, we compared the contribution of individual species to the calculated community metabolic profile (CMP) scores. Well-predicted metabolites were identified by the CMP score model in HPV+H and HPV− groups by examining of the total pool of metabolites with a positive model slope and a model *p*-value < 0.1 (for the explanation see Materials and Methods Section 5.4). Within these groups, we did not find any differences between CERV and VAG (we found the same metabolites), which is in agreement with metagenomic analyses of structure and composition between HPV+H in vaginal and cervical samples (Figure 1 and Figure 2) and as previously described in a bigger sample size [32]. Thus, MIMOSA2 predicted that acetate, proline, and threonine in HPV+H groups and succinate in HPV− groups could be microbiome-derived (Figure 3, Supplementary Table S2). 

### 2.4. Integration of 16S Community Profiling with Urine Metabolome Identified Taxa Associated with Enzymatic Reactions in HPV+H and HPV−

**Figure 4:**
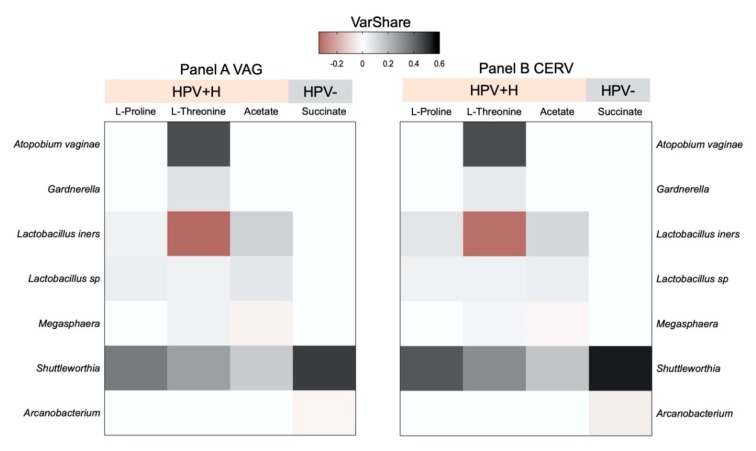
Heatmaps showing the predicted bacterial community-wide urine metabolite turnover in vagina (VAG) and cervix (CERV) associated with the changes in microbiota diversity in HPV+H groups compare to HPV− groups.

Next, we evaluated the metabolic production capacity of a microbial community and key contributor species for well-predicted metabolites (Figure 4) based on genes and reactions and by mapping taxa to correspondent KEGG orthologue (KO) markers. Thus, *Shuttleworthia, Lactobacillus* sp., and *L. iners* were predicted as strong contributors to the reactions of the synthesis of acetate and proline and the degradation of proline and threonine, among other members of the microbial community, in both VAG and CERV HPV+H groups (Figure 4, Supplementary Table S2). Specifically, the role of *Lactobacillus* sp., *L. iners*, and *Shuttleworthia* in the synthesis of acetate, proline, and threonine were predicted on the basis of the following genes: (1) acylphosphatase (K01512), responsible for the synthesis of acetate from acylphosphate; (2) prolyl aminopeptidase (K01259), responsible for the release of N-terminal proline from a peptide; and (3) prolyl-tRNA synthetase (K01881), responsible for the synthesis of tRNAPro and degradation of proline (Figure 4, Supplementary Table S2). Degradation of threonine and synthesis of tRNAThr concomitant with the activity of threonyl-tRNA synthetase (K01868) were positively associated with *Shuttleworthia* and *Lactobacillus* sp., while *L. inners* was a robust negative contributor for this metabolic reaction (Figure 4, Supplementary Table S2). The negative contribution of *L. inners* indicates that it may compete with *Shuttleworthia* and the other *Lactobacillus* species for threonine. *Atopobium vaginae* and *Gardnerella* displayed no associations with acylphosphatase, prolyl aminopeptidase, and prolyl-tRNA synthetase. However, *Atopobium vaginae* was the most potent positive contributor to the degradation of threonine and synthesis of tRNAThr by threonyl-tRNA synthetase, while *Gardnerella* contribution to this reaction was similar to the other species (Figure 4, Supplementary Table S2). In addition, *Megasphaera* was the sole critical negative contributor to the synthesis of acetate from acetyl-CoA predicted based on acetyl-CoA hydrolase (K01067) activity and positive contributor to the degradation of threonine by threonyl-tRNA synthetase, while no other contributions to identified enzymes were found (Figure 4, Supplementary Table S2). 

In contrast, in VAG and CERV HPV− groups, we identified only one positive (*Shuttleworthia*) and one negative (*Arcanobacterium*) predictors associated with the synthesis of cystathionine and succinate by cystathionine gamma-synthase (K01739; Figure 4, Supplementary Table S2).

## 3. Discussion 

The microbiome plays an essential role in the maintenance of human health [34]. However, human microbiome research requires integration with other omic approaches to advance microbiome-based interventions for health and disease management. Pairing metabolomics with metagenomics is now becoming a very promising approach for the identification of complex bacterial metabolic functions, leading towards the development of therapeutic strategies for manipulation of the microbial metabolism in those at risk of disease development [20,21,22]. Taxonomic shifts triggered by different physiological and pathological conditions could modify interactions between microbial community members. Thus, if a competitively dominant species become sensitive to changes in the environment, then subordinate species become beneficiaries from the disturbance and compete with it, interfering with the effects of the dominant species and promoting functional metabolic shifts [35]. Interestingly, by pairing 16S community profiling with urine metabolome, we showed that microbially-produced metabolites explained the dynamic shifts observed in peptide and amino acid metabolism, supporting results from previous studies [21,36]. Microorganisms colonizing the cervicovaginal microenvironment produce a broad range of metabolites that are important for genital health. However, the relationship between taxonomic and functional shifts of the host’s microbiome and peripheral site metabolomes remains largely unexplored, and only a few studies so far have characterized the associations of individual taxa with circulation metabolome using plasma [27] and serum [25,26]. Nevertheless, a recent study aimed to identify taxonomic and metabolic alterations in the cervicovaginal microbiome and urine metabolome in women at risk of preterm premature rupture of the membranes found functional associations between microbiome and metabolome, which could facilitate an appropriate response to a given treatment regime [31]. Therefore, we investigated whether differences in the vaginal and cervical microbiota, in connection to the HPV status, explained variations observed in the urine metabolome dataset. The cervicovaginal community profiles of this sample subset recapitulated results from a previous study [32]. No significant differences among CERV and VAG communities were found (same compositional profiles in both body sites) as community structure did not differ significantly between HPV− and HPV+H; however, VAG HPV+H samples had significantly higher alpha diversity. 

It is recognized that the healthy vaginal microbiome is enriched with *Lactobacillus* sp. with the prevalence of *L. iners* in Hispanics with low-risk HPV and HPV−. We found a taxonomic shift of the cervicovaginal environment in HPV+H groups via a reduction of *L. iners* and an increment in alpha diversity. These findings were also found in another study with dominance in *L. iners* and *L. acidophillus* in healthy women [37]. Moreover, the shift in microbiota diversity due to an HPV+H infection was associated not only with a significant reduction in *L. iners,* but also with the reduction of *Shuttleworthia* species abundances and increase in other *Lactobacillus* sp. in both HPV+H groups. Our results suggest that HPV+H infections could induce changes in physicochemical properties of the genital tract through which niche partitioning may occur and favor the dominance of other species including *Lactobacillus* sp. and a significant reduction in *L. iners* and *Shuttleworthia*. Changes in the diversity of microbial species in the cervical niche could influence the capacity of new community members to produce or consume metabolites. We found a total of 24 urinary metabolites to which we related to the microbiota. In particular, three of these metabolites, i.e., acetate, proline, and threonine, were predicted to be produced by the microbiota in HPV+H groups, which may influence systemic health effects in women at risk of developing cervical cancer. Thus, this taxonomic shift predicted the activity of acetate synthesizing enzyme, acylphosphatase (K01512). The specific role of this enzyme is still not completely elucidated, and limited information is available about its function. Nevertheless, acylphosphatase is known to participate in glycolysis and pyrimidine biosynthesis [38]. Moreover, it is known that the main HPV oncoproteins associated with cervical cancer can favor an increase in the activity of glycolytic enzymes [39]. Thus, the activity of acylphosphatase associated with changes in bacterial composition, including *Lactobacillus* sp. and *Shuttleworthia* in HPV+H groups, could lead to the development of cervical cancer. 

A healthy cervicovaginal environment consists mostly of low-proliferating or quiescent cells. The taxonomic shift likely induced by HPV infections might reprogram cellular metabolism, provoking a “new” metabolic requirement to support uncontrollable cellular proliferation and enhancing protein synthesis. A reduction of populations of *Shuttleworthia* and *L. iners* and an increase in *Lactobacillus* sp. in HPV+H groups also contributed positively to the activity of prolyl aminopeptidase (K01259), which is one of the critical enzymes that control the cell cycle division. This enzyme is still not considered as the target for antitumor therapies. However, recently, it was reported that inhibition of prolyl aminopeptidase could be a targeted treatment strategy in myeloma [40]. Moreover, an identified shift in the contents of *Shuttleworthia*, *L. iners*, and *Lactobacillus* sp. was positively associated with the degradation of proline and synthesis of tRNAPro by prolyl-tRNA synthetase (K01881), while changes in the composition of *Shuttleworthia*, *Lactobacillus* sp., *Atopobium vaginae, Megasphaera*, and *Gardnerella* positively predicted the degradation of threonine and the synthesis of tRNAThr by threonyl-tRNA synthetase (K01868) (Figure 4). In addition, *Lactobacillus* was the dominant taxa, with *L. iners* reducing its abundance in HPV+H accompanied by an increase in Actinobacteria (*Atopobium vaginae*). *Atopobium vaginae* is a marker for bacterial vaginosis [41] and other pelvic complications such as endometritis, ovarian abscesses, and preterm delivery [42,43,44]. This bacterium was also found in association with cervicovaginal samples, both HPV+H and low-risk HPV [32,45], and in this study, we identified it to be a most potent positive contributor to the degradation of threonine and the synthesis of tRNAThr by threonyl-tRNA synthetase as well as the degradation of proline and the synthesis of tRNAPro by prolyl-tRNA synthetase. Indeed, our results confirmed those of a recent paper on metabolic profiling of cervicovaginal lavages, where *A. vaginae* were reported to be associated with the metabolism of l-threonine [21]. Prolyl-tRNA synthetase and threonyl-tRNA synthetase are members of the aminoacyl-tRNA synthetases family, participating in the process of charging a cognate amino acid to tRNA. However, aminoacyl-tRNA synthetases are not only known for their canonical role in protein synthesis but are also involved in a variety of non-canonical processes [46]. Therefore, targeting aminoacyl-tRNA could emerge as a strategy to not only combat cancer but also for managing microbial [47] or parasite infections [48]. Recent reports confirm the suitability of aminoacyl-tRNA as a promising target for cancer therapy, showing that inhibition of prolyl tRNA synthetase activity in an SK-MEL-2 human melanoma cell line suppresses their proliferation [49], while overexpression of threonyl-tRNA synthetase is correlated with the progression of human ovarian cancer [50]. 

In both HPV+H groups, *Megasphaera* was the only one negative contributor to the synthesis of acetate from acetyl-CoA associated with the activity of acetyl-CoA hydrolase (K01067), an enzyme that participates in pyruvate metabolism catalyzing the hydrolysis of acetyl-CoA. The negative contribution may indicate an antagonistic relationship between microbes and competition for the substrate in the same niche [51]. As a result, the metabolic effects produced by the other community members are mitigated. We suggest that the predicted effect produced by *Megasphaera* could be associated with the change of pyruvate carbon flux towards the release of lactate, rather than conversion to acetyl-CoA. In this case, acetyl-CoA could be further metabolized to CoA and acetate and eventually lead to the malignant transformation of the cervical cells [39]. 

In contrast to HPV+H groups, in HPV− groups, we identified that only two low-dominant members of the microbial community, *Shuttleworthia* and *Arcanobacterium,* could predict the production of cystathionine and succinate from cysteine by cystathionine gamma-synthase (K01739), either positively (*Shuttleworthia)* or negatively (*Arcanobacterium).* These data suggest that *Shuttleworthia and Arcanobacterium* were competing for cysteine. We speculate that this competition is required to support a physiological, metabolic balance between anabolic and catabolic reactions in the cervicovaginal environment of HPV− groups. 

Overall, the cervicovaginal microbiota seems to regulate the differential synthesis and degradation of amino acids with possible roles in the inflammatory and cell-cycle outcomes, suggesting that these biochemical gradients may result in niche partitioning and physiological outcomes in this infectious context. Indeed, species sharing the same cervical habitat (both dominant and rare taxa) have similar biochemical needs and may use resources in somewhat different ways to avoid direct competition, thus devising a certain niche partition, with more direct competition among the low dominant taxa. These results suggest that metabolite-driven niche partitioning may be an important factor in the maintenance of high microbial diversity in HPV+H infected cervix, although additional studies using cervical lavage metabolomics will add more information as to the specific metabolisms of cervical bacteria during HPV infections. 

We also cannot discard the relationship between metabolites and microbiota not detected in our study due to low power. Our study provides only a partial view of the metabolite-driven niche partitioning and potential metabolic interactions among these microbes and urine level metabolites in different HPV infections. Metabolite diversity must contribute to niche differentiation and support diversity due to substrate preferences among heterotrophs. However, given that this data relates urine-based metabolites with HPV infections status and concomitant cervical microbiota, it calls for more niche directed studies to be conducted. Indeed, we anticipate that the addition of other samples such as cervical lavages and an increase in the sample number would be required to support general conclusions about ecological niche partitioning in the cervix considering HPV status and metabolic profiling. 

## 4. Conclusions

The functional characterization of vaginal and cervical HPV+H, HPV−, microbiota, and urinary metabolome via integration of multi-omics approaches showed specific differences in microbiome/metabolome associations. We identified species that can predict the production or degradation of key metabolites, either positively or negatively, contributing to the pool of circulating urinary metabolites. However, since MIMOSA2 is a tool for hypothesis generation, further validation studies would be needed to confirm whether the taxa and genes in question are modifying the metabolites according to the predictions. We also recognize that other confounding effects, such as inter-individual variation and diet, can be a limitation when analyzing serum and urine metabolomic levels. Moreover, further investigations are required using larger patient cohorts, including samples with cervical neoplasia or cancer and application of different analytical platforms to validate our finding or identify the other metabolites/microbiota relationships that are beyond the scope of this study. Nonetheless, our findings that HPV+H and HPV− cervicovaginal microbiomes and urinary metabolomes were functionally separated through niche partitioning offers additional information on the complex systemic interactions of the microbiome in human health. Taken together, analysis of functional coupling of cervical microbiome/host urine metabolome predicts the occurrence of specific metabolic abnormalities that could lead to cervical cancer development.

## 5. Materials and Methods 

### 5.1. Patient Recruitment, Sampling Acquisition, and Human Papilloma Virus Genotyping

We selected 19 samples (cervicovaginal and urine) corresponding to 8 HPV− and 11 HPV+H (HPV low risk were not considered) from the analyses of 62 patient samples from women coming for gynecology evaluation at the University of Puerto Rico and San Juan City clinics (San Juan Metropolitan area). Samples were obtained under the Ethics Committees of the UPR-Medical Sciences Campus IRB (Protocol ref. 1050114), described in Godoy-Vitorino et al. (2018a). Cervical and vaginal samples were collected by the ob/gyn after informed consent procedures, and HIPAA forms were signed in accordance with the Declaration of Helsinki. Besides swabs, urine biofluid was self-collected at the time of gynecology evaluation. All samples were stored at −80 °C and processed for further genomic DNA and metabolite extractions. HPV genotyping was done with Labo Bio-Medical Products (LBP; Rijswijk, The Netherlands, licensed Innogenetics technology) as previously described [2,32]. The selected samples corresponded to 11 HPV+H and 8 HPV− samples. The correspondent metadata was previously published [32]. 

### 5.2. Microbiota Analyses

16S rRNA genes were analyzed using the Quantitative Insights into Microbial Ecology (QIIME) v1.9 pipeline [52]. The Greengenes database [53] was used to identify operational taxonomic units (OTUs) with 97% sequence similarity. Sequence data was retrieved from SRA BioProject accession #PRJNA429969. Alpha diversity, represented by the Chao1 index, explains species richness (OTU count) that can be detected in a microbial ecosystem, and beta diversity represents differences in the microbial structure, taking into account species composition in one environment compared to another (plots show how similar are samples on basis of its composition). Alpha diversity analyses were plotted via rarefaction curves using the Chao1 index, and beta-diversity was plotted with PCoA. The metadata of these selected samples is present in Supplementary Table S1. 

### 5.3. Metabolomics Analysis

Two hundred μL of liquefied urine samples were collected and used for metabolite extraction, derivatization, and fractionation by gas chromatography-mass spectrometry (GC-MS) (GCMS-QP2010, Shimadzu Scientific, Columbia, MD, USA), as described before [32]. Mass spectral library searches of the major chromatographic peaks were conducted using the GCMS Lab solution data analysis software (Shimadzu) equipped with the NIST14/2014/EPA/NIH database. Twenty-four metabolites were identified [32] and subjected to metabolomics analysis using Metaboanalyst.ca [54]. A total of 157 peaks were initially detected in the urine samples. Peak integration for all metabolites was performed using GCMS Labsolution data analysis software (Shimadzu) equipped with the NIST14/2014/EPA/NIH database in each data set followed by extensive mass spectral library searches of the major chromatographic peaks, which resulted in a final data set consisting of twenty-four metabolic features selected for the metabolomics analysis. Reproducibility of metabolite recovery, the performance of sample extraction, derivatization, and instrumentation were validated by the utilization of several blank samples, including a system suitability blank, extraction processing blank, and derivatization processing blank. To evaluate analytical accuracy and precision, we performed an external quality assessment using 2-Fluobiphenyl (Sigma-Aldrich, St. Louis, MO, USA) as quality control (QC) samples. 2-Fluobiphenyl was spiked into derivatization blank samples before running on the GC/MS at different concentrations (*n* = 6). The percent of relative standard deviation (%RSD) of 2-fluobiphenyl peak abundance in the QC samples (*n* = 6) was calculated using GCMS Labsolution data analysis software (Shimadzu). Thus, %RSD for peak abundances of QC samples accounted for 4.6%, which demonstrates good reproducibility of the method. To mitigate systematic bias, we performed the randomization of the sample analysis order. Blanks and QC samples were spaced evenly among the injections to monitor instrument stability. Quality control during data processing was previously described in detail [32]. In our previous study, we did not identify any metabolic differences in urine metabolome in women diagnosed simultaneously with HPV+H, CIN1, or CIN3 lesions [32]. Preliminary analyses performed considering low-grade and high-grade lesions did not identify any metabolite–microbiota associations that passed the selected criteria of the MIMOSA2 model. Thus, our current follow-up study is focused only on relating the metabolome–microbiota associations using patient samples with or without HPV infection status.

### 5.4. Bioinformatic Analysis using Multi-Omics Integration of Metabolome and Microbiome 

Integration of metagenomics and metabolomics data were performed using Model-based Integration of Metabolite Observations and Species Abundances 2 (MIMOSA2), freely available at borensteinlab.com/software_MIMOSA2.html [33]. MIMOSA2 summarizes paired microbiome–metabolome datasets to support mechanistic interpretation and hypothesis generation. MIMOSA2 applies a method for predicting relative metabolic turnover, using a metabolic network model to translate the resulting enzymatic gene abundance estimates into community-based metabolite potential (CMP) scores. Moreover, MIMOSA2 characterizes the relative capacity of community members to produce or consume metabolites based on a priori metabolic information of the activity of metabolic enzymes for each species from the KEGG database, describes how well each metabolite can be predicted by metabolic potential, and estimates how much each taxon can explain each metabolite. While correlation-based statistical analyses of metabolomic measurements are not mechanistic, this framework has the advantage of proposing mechanisms for the contributions of species to the turnover of particular metabolites. A more detailed description of this framework can be found in previously published work by the developers [33]. However, the current version of MIMOSA2 has several limitations, including the inability to capture host metabolism, and it does not consider signaling processes, transcriptional regulation, or bounds on metabolic fluxes. Nevertheless, it assigns effects for enzymes catalyzing nonreversible reactions and presumably captures major metabolic fluxes for well-characterized microbes, but the information is lost from reversible reactions, which may hinder the prediction of metabolites in other pathways [33]. 

Greengenes OTUs taxonomic abundances of VAG and CERV microbiomes and a host metabolome previously identified in urine [32] were used for calculation of CMP scores for each metabolite and sample and their functional characterization. The following options were specified on the MIMOSA2 input interface: (1) Microbiome data—Greengenes 13_5 or 13_8 OTUs, (2) gene content and metabolic model source—PICRUSt and KEGG model, and (3) similarity threshold for mapping ASVs to OTUs—0.99. OLS regression was used for the identification of well-predicted metabolites by MIMOSA2. Metabolites were normalized by log-transformation and Pareto scaling, as published before [32]. Well-predicted metabolites were identified by the CMP scores model in HPV+H and HPV− groups by examining of the total pool of metabolites with positive model slope and a model *p*-value < 0.1. Given that MIMOSA2 is prone to false negatives because its model is approximate, it requires a linear/monotonic relationship between metabolite abundances and inferred metabolic effects of the microbiome based on genes or taxa. Therefore, developers of MIMOSA2 suggest using a weak statistical threshold (*p*-value < 0.1) to capture more relationships of possible interest, where the data might be only partially described by the approximate model but might still suggest some underlying biological signal. 

Metabolic capabilities of microbial taxa and variation in microbiome composition paired to both metabolite measurements and information from the KEGG database were used to estimate the metabolic capabilities of individual species identified in VAG and CERV microbiota by metagenomics. The heat map depicts microbial features that may underlie differences in microbial metabolite concentrations between similar communities. It was constructed using values that represent the fraction of the variation (VarShare) in each metabolite explained by the taxon in question, according to the overall community model (Supplementary Table S2). To note, OTU 227000, named *Shuttleworthia* in the Greengenes database and in our manuscript, has been proposed to be BVAB1 [33].

## Supplementary Materials

The following are available online at https://www.mdpi.com/2218-1989/10/1/36/s1, Table S1: Samples groups mapping, Table S2: Contribution results.
